# An Automated Quantification Tool for Angiogenic Sprouting From Endothelial Spheroids

**DOI:** 10.3389/fphar.2022.883083

**Published:** 2022-04-27

**Authors:** Pavitra Kannan, Martin Schain, David P. Lane

**Affiliations:** ^1^ Department of Microbiology, Tumor and Cell Biology, Karolinska Institutet, Stockholm, Sweden; ^2^ Antaros Medical AB, Mölndal, Sweden

**Keywords:** sprouting angiogenesis, automated quantification, spheroids, *in vitro* angiogenesis assay, sprouting parameters

## Abstract

The process of sprouting angiogenesis can be measured *in vitro* using endothelial cells in sprouting assays such as the fibrin bead assay and the spheroid-based assay. While the technical aspects of these sprouting assays have been well-optimized, the analysis aspects have been limited to manual methods, which can be time-consuming and difficult to reproduce. Here, we developed an automated analysis tool called AQuTAS to quantify sprouting parameters from the spheroid-based sprouting assay. We trained and validated the algorithm on two subsets of data, and tested its sensitivity by measuring changes in sprouting parameters over a range of concentrations of pro- and antiangiogenic compounds. Our results demonstrate that the algorithm detects known differences in sprouting parameters in endothelial spheroids treated with pro- and antiangiogenic compounds. Moreover, it is sensitive to biological changes that are ≥40%. Among the five quantified parameters, cumulative sprout length is likely the most discriminative parameter for measuring differences in sprouting behavior because it had the highest effect size (>1.5 Cohen’s d). In summary, we have generated an automated tool that quantifies sprouting parameters from the spheroid-based assay in a reproducible and sensitive manner.

## 1 Introduction

The formation of new blood vessels from existing ones, known as sprouting angiogenesis, is a key step in the development of cancer (Potente et al., 2011). In response to growth factors or hypoxic conditions, endothelial cells on existing vessels begin migrating toward the growth signal and form a new vascular sprout. Over time, this sprout becomes a capillary and forms a lumen in which blood can flow. The ability to study this process in cancer development using relevant experimental systems is instrumental for identifying new ways to hinder or inhibit sprouting angiogenesis during cancer progression (Nowak-Sliwinska et al., 2018).

Experimental systems commonly used to study sprouting angiogenesis in 3D are the fibrin bead assay and the spheroid-based assay. In the fibrin bead assay, endothelial cells are seeded onto a dextran-coated microcarrier bead and embedded into a fibrin gel. Over the course of several days, the endothelial cells sprout and form capillary-like structures with a lumen (Nakatsu et al., 2007; Nakatsu and Hughes, 2008). In the spheroid-based assay, endothelial cells are seeded as spheroids and embedded in collagen or fibrin gel. Unlike in the fibrin bead assay, endothelial cells in the spheroid-based assay form only partially lumenized sprouts (Heiss et al., 2015; Tetzlaff and Fischer, 2018; Favara et al., 2021; Santos et al., 2017). The fibrin bead assay has even been adapted to 96-well plates, providing the possibility for high-throughput drug screening of anti-angiogenic compounds (Eglinger et al., 2017; Carpentier et al., 2020); no such adapted format exists for the spheroid-based assay.

While the technical aspects of sprouting assays have been optimized (Nakatsu et al., 2007; Nakatsu and Hughes, 2008; Nehls and Herrmann, 1996; Newman et al., 2011), improvements in the automated analysis of sprouting angiogenesis have been fairly limited. For both the fibrin bead assay and the spheroid assay, analysis has traditionally been performed manually for individual sprouts, which can be time consuming and difficult to reproduce. To address these issues, several groups have recently developed automated analyses to quantify angiogenic sprouting from the fibrin bead assay using ImageJ, including the Sprout Morphology (Eglinger et al., 2017) and Angiogenesis Analyzer (Carpentier et al., 2020) plugins.

However, no automated analysis tool exists for the spheroid-based sprouting assay. Existing algorithms for fibrin bead sprouting applied to the spheroid-based assay are likely to perform poorly because they depend on detection of a circular bead in the image to separate sprouts from the bead. In addition, due to pandemic-related shortages, microcarrier beads have not been available to perform the fibrin bead assay. Thus, there is a need for automated quantification of angiogenic sprouting for the spheroid-based assay.

Here, we present an automated algorithm called AQuTAS that measures angiogenic sprouting parameters from the spheroid-based assay. To validate the algorithm, we compared automated measurements to manual measurements and tested the algorithm on two datasets. To measure the sensitivity of the algorithm, we also quantified sprouting parameters under a range of concentrations of pro- and anti-angiogenic compounds. The algorithm, built in Matlab (with the code available for download), batch processes hundreds of spheroid sprouts within minutes, enabling quantification of angiogenic sprouting from the spheroid-based assay.

## 2 Materials and Methods

### 2.1 Reagents

Reagents for the sprouting assay were prepared using previously described protocols (Nakatsu et al., 2007; Eglinger et al., 2017) but with the following modifications. Thrombin (T4648-1KU, Sigma Aldrich) was dissolved in sterile water at 50 U/mL, while aprotinin (A-1153, Sigma Aldrich) was dissolved in 4 U/mL distilled water and sterile filtered. Aliquots were stored at −20°C until use. Fibrinogen type 1 (F-8630, Sigma Aldrich) was prepared fresh before each experiment: 6 mg/ml fibrinogen was dissolved in basal Endothelial Growth Medium 2 (EGM-2, Promocell), warmed to 37°C for 10 min, and sterile filtered (0.20 μm). A 1.2% (w/v) methylcellulose solution was made by dissolving 4,000 cP methylcellulose (M01512, Sigma Aldrich) in basal EGM-2 and stored at 4°C. Recombinant Vascular Endothelial Growth Factor 165 (0.5 ng/ml, Promocell) was used to induce angiogenesis [(Ferrara and Henzel, 1989), while sunitinib malate (dissolved in DMSO, SU11248, SelleckChem) was used to inhibit angiogenesis (Sun et al., 2003)].

### 2.2 Cell Culture

Human umbilical vein endothelial cells from pooled donors (HUVECs, Promocell) were used for sprouting experiments. Upon receipt of the vial, 1 × 10^6^ cells were expanded for one passage according to manufacturer’s instructions, checked for absence of mycoplasma (MycoAlert Mycoplasma Detection Kit, Lonza), and frozen down in aliquots of 2 × 10^5^ cells in cryofreezing medium (Promocell). HUVECs were cultured from passages 2 to 6 in EGM2 medium comprising 2% fetal calf serum, epidermal growth factor (5 ng/ml), basic fibroblast growth factor (10 ng/ml), insulin-like growth factor (20 ng/ml), vascular endothelial growth factor 165 (0.5 ng/ml), ascorbic acid (1 μg/ml), heparin (22.5 μg/ml), and hydrocortisone (0.2 μg/ml), henceforth referred to as complete medium. DetachKit2 (Promocell) was used for detachment. Cells were maintained in an incubator at 37°C, 5% CO_2_, and 21% O_2_, and culture medium was replaced every 2–3 days.

### 2.3 Generation of Human Umbilical Vein Endothelial Cells Spheroids

Three to 4 days prior to generation of HUVEC spheroids, cells were seeded at a density of 1 × 10^4^ cells/cm^2^ in a T-25 flask (TPP) and were allowed to reach 80–90% confluency before use; near-confluent cultures were necessary to obtain visible sprouting. On the day of spheroid seeding, HUVECs were fluorescently labelled with 5 μM CellTracker Green 5-chloromethylfluorescein diacetate (CMFDA, Invitrogen) in EGM-2 basal medium (serum-free, no added components) for 20 min at 37°C. Cells were then harvested using the DetachKit2 (Promocell) according to manufacturer’s instructions, counted, and resuspended in complete EGM-2 medium containing 40% methylcellulose solution (v/v). HUVEC spheroids were then generated using the hanging drop method (Korff and Augustin, 1998; Heiss et al., 2015; Tetzlaff and Fischer, 2018). Briefly, 20 μL drops of the cell solution were pipetted on the lid of a 10 cm dish (TPP). The dish was then inverted, and the bottom was filled with 7 ml PBS. The hanging drops were incubated for 24 h before use. For four wells of spheroids, 7.5 × 10^4^ cells were resuspended in 2.4 ml of medium and 0.6 ml of methocel; this ratio was scaled as needed to seed the required amount of wells.

### 2.4 Initiation of Sprouting Assay

Sprouting assays were performed by embedding HUVEC spheroids into fibrin gel, using a modified protocol from that previously reported (Santos et al., 2017). HUVEC spheroids were collected in basal EGM-2 medium, pelleted for 1 min at 50 × g, and resuspended in solution comprising 60% (v/v) fibrinogen solution (6 mg/ml stock diluted in basal EGM-2 to achieve 2.3 mg/ml final concentration in each well) and 40% (v/v) methylcellulose (1.2% solution adjusted in volume using basal EGM-2). The volume of solution was calculated based on the number of wells used (90 μL/well), resulting in an approximate concentration of 250 spheroids/mL. Aprotinin (diluted to 0.15 U/mL final concentration) was added directly to the resuspension solution. After pipetting 10 μL of thrombin (diluted to 0.625 U/mL final concentration in basal EGM-2) to the target wells of a pre-warmed, black 96-well plate with clear bottom (Corning 3603), 90 μL of the spheroid solution was added dropwise to each well and gently mixed with a pipette three to four times. The plate was left in the hood for 5 min before being transferred to an incubator for 60 min to solidify. Complete EGM-2 medium was then added dropwise to each well (150 μL/well) containing the indicated concentrations of VEGF (0, 5, 10, 20, 40 ng/ml) or sunitinib malate (0, 0.01, 0.1, 1, 10 μM).

### 2.5 Imaging of Angiogenic Sprouts

Images were acquired using a Cytation 5 multi-mode microplate reader (Biotek). The turbidity of the gel in each well was measured by scanning absorbance at 350 nm. Each well was then imaged at 37°C and 5% CO_2_ by phase contrast microscopy and by fluorescence microscopy using a 4× objective (Olympus UPLFLN 4 × Ph, NA 0.13). For fluorescence imaging, a 465 nm LED source was used with an excitation wavelength of 469/35 and an emission wavelength of 525/39. Each well was imaged using the Z-stack and montage features of the Cytation 5 software, resulting in final image size of 1973 μm × 1457 μm in x and y, and 12 slices in z with a step of 54 μm. Autofocus was performed based on the objective size. Whole-well images were stitched together using Gen5 software and downsampled by 20%, resulting in a stitched image with a 4 × 4 mm coverage (19 Mb in tiff format) and a resolution of 2 μm per pixel for each z-plane that was exported for image analysis.

### 2.6 Automated Image Analysis

#### 2.6.1 Generation of Masks

Sprouting parameters were measured using a custom algorithm written in MATLAB R2018b (v. 9.5.0.9.44444, MathWorks Inc.). Images of single sprouts were first cropped using the inbuilt function *imcrop* and saved as individual files in tiff format. Masks of the total spheroid area were generated as previously described (Kannan et al., 2016). Briefly, images were first processed using a median filter of 1 × 1 pixels to remove electronic noise using *medfilt2* function and contrast enhanced using the *adapthisteq* function, which transforms values using an adaptive histogram equalization process. Edges were then segmented using the Sobel method, dilated using a 2D convolution between the edge mask and the width of the Gaussian kernel (100), and thresholded to remove background pixels. For each image, the threshold was defined as the value corresponding to the median value of all nonzero elements +0.3* standard deviation value of all nonzero elements, which was empirically determined. The spheroid center was identified on a smoothened image (median filter, 10 × 10) using the brightest pixels and expanded using the *imdilate* function. The sprout area was identified by subtracting the spheroid center from the total spheroid area.

#### 2.6.2 Sprout Skeletonization

The parameters migrated sprouts, attached sprouts, and cumulative sprout length were subsequently calculated from a skeletonized sprout. An initial sprout skeleton was generated by applying the *bwskel* function (Lee and Kashyap, 1994) on the sprouting area, using a minimum branch length of 1 pixel. After the spheroid center was subtracted from the skeleton, the remaining skeleton was dilated using a diamond structural element of radius 2 pixels, pruned using the *bwskel* function, and filtered using the *bwconncomp* function with a threshold of 6 to remove small (“noisy”) extensions. To identify whether sprouts were still attached to the spheroid body or had migrated on the focal plane, we measured whether each sprout intersected with the edge mask of the center; sprouts that did not intersect with the center on the focal plane were labeled “migrated” while those that intersected were labeled “attached”. Due to limitations in the objective’s focal plane, we could not determine whether the “migrated” sprouts were attached to the spheroid on a different Z plane. Maximum intensity projections also could not be used because fluorescence emitted from the spheroid body resulted in a halo-like effect around the entire spheroid sprout. The cumulative sprout length was calculated as the sum of all pixels detected in the final sprout skeleton.

#### 2.6.3 Accessing and Running the Algorithm

Users have two main options for accessing the algorithm. Users without a MATLAB license can run the tool using the standalone application, available for download as the folder “AQuTAS Standalone” (Zenodo repository: 10.5281/zenodo.6444392). Prior to using the tool, users must install MATLAB Runtime by following the instructions in the document “Instructions Install Standalone.txt”, contained in the downloaded folder. Users with a Matlab license can run the tool using the GUI (executable through AQuTAS.fig file), available for download as the folder “AQuTAS Matlab” (Zenodo repository: 10.5281/zenodo.6444392).

The algorithm can then be run by following the document “Instructions Run AQuTAS.txt”, contained in either of the downloadable folders. Before running any of the other features of the tool, the user must first add the folder containing code files to the tool. This is done by selecting the “Select folder with code” button of the GUI. Next, to perform quantification, the user must ensure that the folder for analysis contains images of individual spheroids. If images are to be cropped, the user should click on the “Crop single images” button of the GUI. Afterwards, the user should click on the “Analyze sprouts (FL)” button of the GUI and select the folder containing images of individual spheroid sprouts. The algorithm should then process all the images of the entire dataset. Output parameters (total spheroid area, total sprouting area, total number of sprouts including migrated and attached, and cumulative sprout length) for all images are generated in an output csv file. Source code is also available at Github (https://github.com/p-kannan/aqutas).

### 2.7 Manual Image Analysis

Three sprouting parameters (total spheroid area, cumulative sprout length, and total number of sprouts) were measured manually using FIJI (v. 1.52 h, ImageJ). Cropped images of spheroid sprouts, saved as individual files in tiff format, were smoothened using a median filter of 1 × 1 and thresholded using the Triangle method (with dark barkground) to generate the spheroid mask. Cumulative sprout length and total number of sprouts were measured by manually tracing sprouts on the original spheroid image using the Simple Neurite Tracer plugin (Longair et al., 2011).

### 2.8 Comparison With Existing Quantification Methods

To test our assumption that existing algorithms for fibrin bead sprouting applied to the spheroid-based assay would perform less well than AQuTAS, we compared the skeletonization of spheroid sprouts generated by AQuTAS to that generated by two existing algorithms for fibrin bead assay, Angiogenesis Analyzer and Sprout Morphology. For Angiogenesis Analyzer (Carpentier et al., 2020), default settings were used for the “Fluorescence” module. For Sprout Morphology (Eglinger et al., 2017), we used the following parameters to detect the spheroid center: the Triangle method for thresholding, 0.06 for blur radius of bead detection, 33 pixels for minimum bead radius, and one as the factor for dilation of beads. To detect sprouts, we used the following parameters: Triangle method for thresholding, 0.04 blur radius, 1,000 minimal area of plexus, and 10 minimal sprout area.

### 2.9 Statistical Analysis

Data used for the optimization of the algorithm were checked data for homogeneity of variance and then evaluated for statistical significance using a t-test with Welch’s correction (unpaired, two-tailed, *α* = 0.05) or using a Mann-Whitney test (non-parametric, unpaired, two-tailed, alpha = 0.05). Data used in the comparison of automated vs. manual results were fit with linear regression models. Data used in evaluating the sensitivity of the algorithm to pro- and anti-angiogenic compounds were evaluated for statistical significance using a Browne-Forsythe one-way ANOVA, followed by multiple testing correction using Dunnett T3 (two-tailed, *α* = 0.05). Effect sizes were calculated using Cohen’s d; values < 0.5 were considered small, 0.5 to 1.0 were considered medium, and >1.0 were considered large.

Data points represent individual spheroids from one to three biological experiments, as indicated in the text or figure legend. For experiments involving pro- and anti-angiogenic compounds, sample sizes were estimated from the initial training data set (*β* = 0.8). Although randomization and blinding were not possible, data were analyzed using the automated algorithm, limiting user bias on individual samples.

## 3 Results

### 3.1 Optimization of Spheroid Sprouting Assay in 96-Well Plate Format

To increase the sample sizes needed to optimize the algorithm, we first adapted the spheroid sprouting assay to a 96-well plate format (Eglinger et al., 2017; Carpentier et al., 2020); only the fibrin bead assay had previously been adapted to this format. We tested both fibrin and collagen as matrix components for the spheroid sprouting assay, but ultimately chose fibrin for three reasons. First, fibrin deposition is involved in tumor angiogenesis (Feng et al., 2013). Second, unlike fibrin, collagen was difficult to work with in a small-well format because its high viscosity led to bubble formation during pipetting (Supplementary Figure S1). Third, collagen requires pH adjustment to initiate polymerization, and variations in the pH of the gel influence sprouting behavior (Nehls and Herrmann, 1996). Thus, we chose to optimize the spheroid sprouting assay using fibrin gel as the matrix, where gel components were dissolved in cell medium (pH 7.4) to reduce variation in pH across experiments.

**FIGURE 1:**
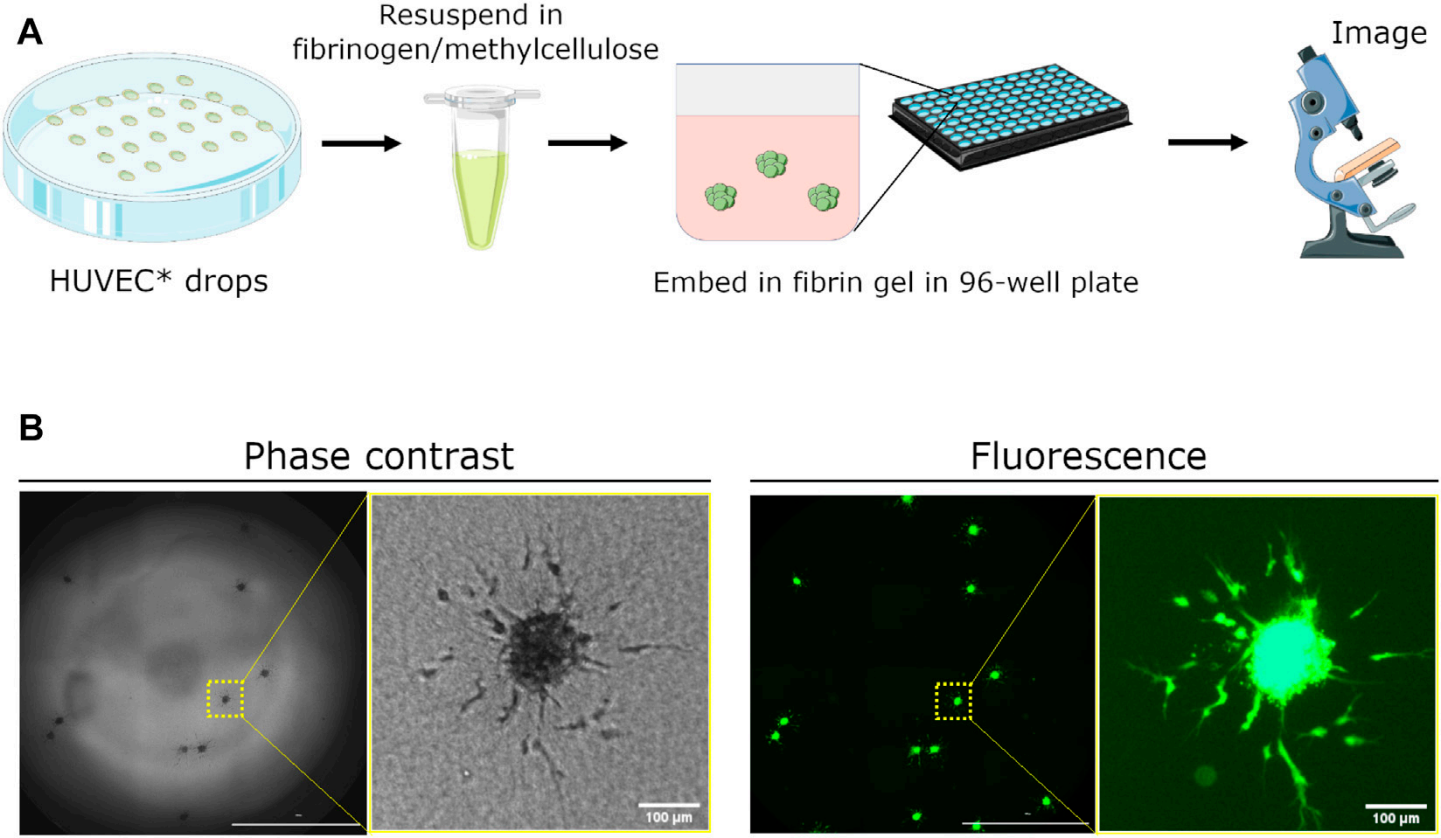
Schematic demonstrating setup of spheroid assay for angiogenesis sprouting. **(A)** Human umbilical vein endothelial cells (HUVEC) are fluorescently dyed with CellTracker Green CMFDA (5 μm) and seeded as hanging drops for 24 h. Spheroids are collected, resuspended in a fibrinogen/methylcellulose solution, and mixed with thrombin in a 96-well plate to embed spheroids within the generated fibrin gel. Medium containing pro- or anti-angiogenic factors is added 1 h later to initiate sprouting. Angiogenic sprouting is imaged 24 h later using a real-time, fluorescence microscopy plate reader. **(B)** Representative images of embedded spheroids in a single well and a close-up of sprouting from a single spheroid. Images were acquired using phase contrast and fluorescence microscopy. Scale bar of whole-well image = 2000 μm.

Human umbilical vein endothelial cells (HUVECs) were fluorescently labeled with CellTracker Green to enhance imaging contrast and to avoid meniscus-related effects visible in phase-contrast images. Once spheroids were formed using the hanging drop method, they were resuspended in a solution comprising 60% of fibrinogen and 40% methylcellulose made in basal EGM-2 medium (Figure 1A). We found that methylcellulose was a crucial to preventing the spheroids from sinking to the bottom of the well (Supplementary Figure S2). The fibrinogen/methycellulose solution containing spheroids was then pipetted into each well pre-containing thrombin to initiate formation of the fibrin gel. Whole wells containing spheroids were then imaged 24 h later using phase-contrast microsocpy and fluorescence microscopy (Figure 1B).

### 3.2 Development of Automated Algorithm to Quantify Angiogenic Sprouting

**FIGURE 2:**
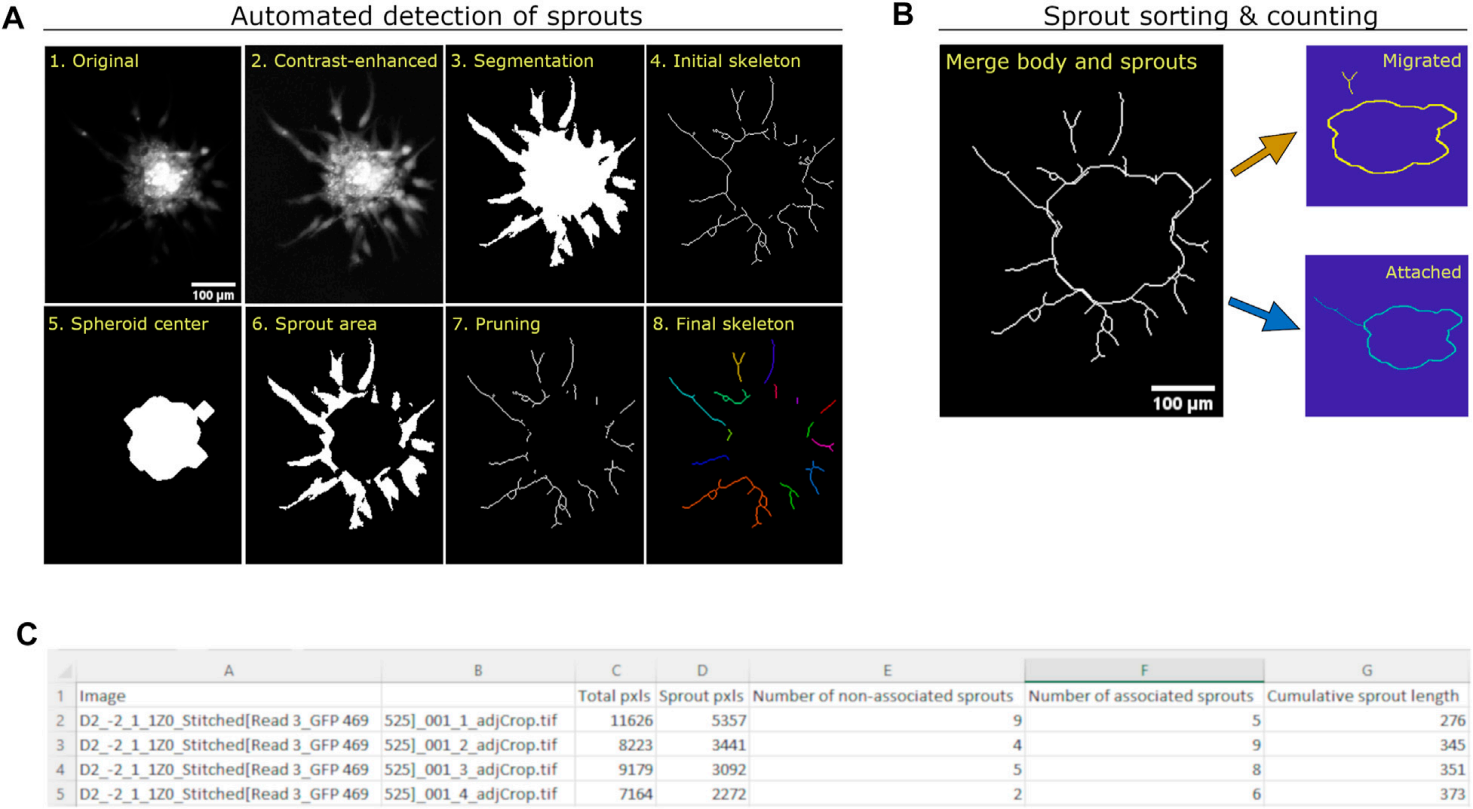
Quantification method of angiogenic sprouting from HUVEC spheroids. **(A)** Images demonstrating the automatic generation of masks and skeletons used to quantify total area, sprout area, and cumulative sprout length from each spheroid. The original image (1) is intensity adjusted using adaptive histogram equalization (2), and segmented using a combination of Sobel segmentation, convolution, and adaptive thresholding to generate a spheroid mask (3), as described before (Kannan et al., 2016). The image was then reduced to an initial skeleton using a skeletonization method (4). After the spheroid center (5) and sprout area (6) were identified, the center was subtracted from the sprout skeleton to exclude extensions found in the spheroid center (7). Small extensions were then removed to generate the final sprout skeleton (8). **(B)** Images demonstrating the counting of individual sprouts as migrated or attached. Each sprout in the skeleton is assessed as to whether it intersects with the skeleton of the center. If a sprout intersects with the center, it is considered “attached”; otherwise, it is considered “migrated”. **(C)** View of output csv file from automated algorithm.

We subsequently developed a Matlab-based automated algorithm to quantify angiogenic sprouting from HUVEC spheroids imaged using fluorescence microscopy (Figure 2). After individual spheroids were cropped out manually from the stitched, whole-well image (Figure 2, panel 1), they were contrast enhanced using an adaptive histogram equalization process to improve visualization of the sprouts (Figure 2, panel 2). The total spheroid area was segmented using edge detection, smoothed using convolution, and automatically thresholded to remove background pixels (Figure 2, panel 3). The total area was used to generate an initial sprout skeleton (Figure 2, panel 4), which is done by reducing the sprouting area to a 2-dimensional line structure based on the image topology. After the spheroid center was identified using the brightest pixels (Figure 2, panel 5), it was removed from the total spheroid area to generate the sprouting area (Figure 2, panel 6) and from the initial skeleton to remove “sprout extensions” located within the spheroid body (Figure 2, panel 7). The sprout skeleton was further pruned to remove small extensions, resulting in a final skeleton for analysis (Figure 2, panel 8). Existing methods designed to quantify sprouting parameters in the fibrin bead assay detected fewer sprouts than our method, likely because previous methods rely on detection of a circular bead that does not exist in the spheroid-based assay (Supplementary Figure S3).

Since pro- and anti-angiogenic compounds could affect migration of endothelial cells differently, we sorted identified sprouts into two sets. Some of the spheroid sprouts were still attached to the spheroid body and were therefore labeled “attached”, while others appeared to have detached from the body and were labeled “migrated”. Since the images used for analysis were obtained from only one focal plane, we could not determine if the migrated sprouts were in fact endothelial cells that had migrated or if their attachment point was only visible from a different imaging plane (Figure 2B). Results of the quantification parameters (total spheroid area, total sprouting area, total number of sprouts including migrated and attached, and cumulative sprout length) are provided for all the images in an output csv file (Figure 2C).

### 3.3 Validation of Automated Algorithm

**FIGURE 3:**
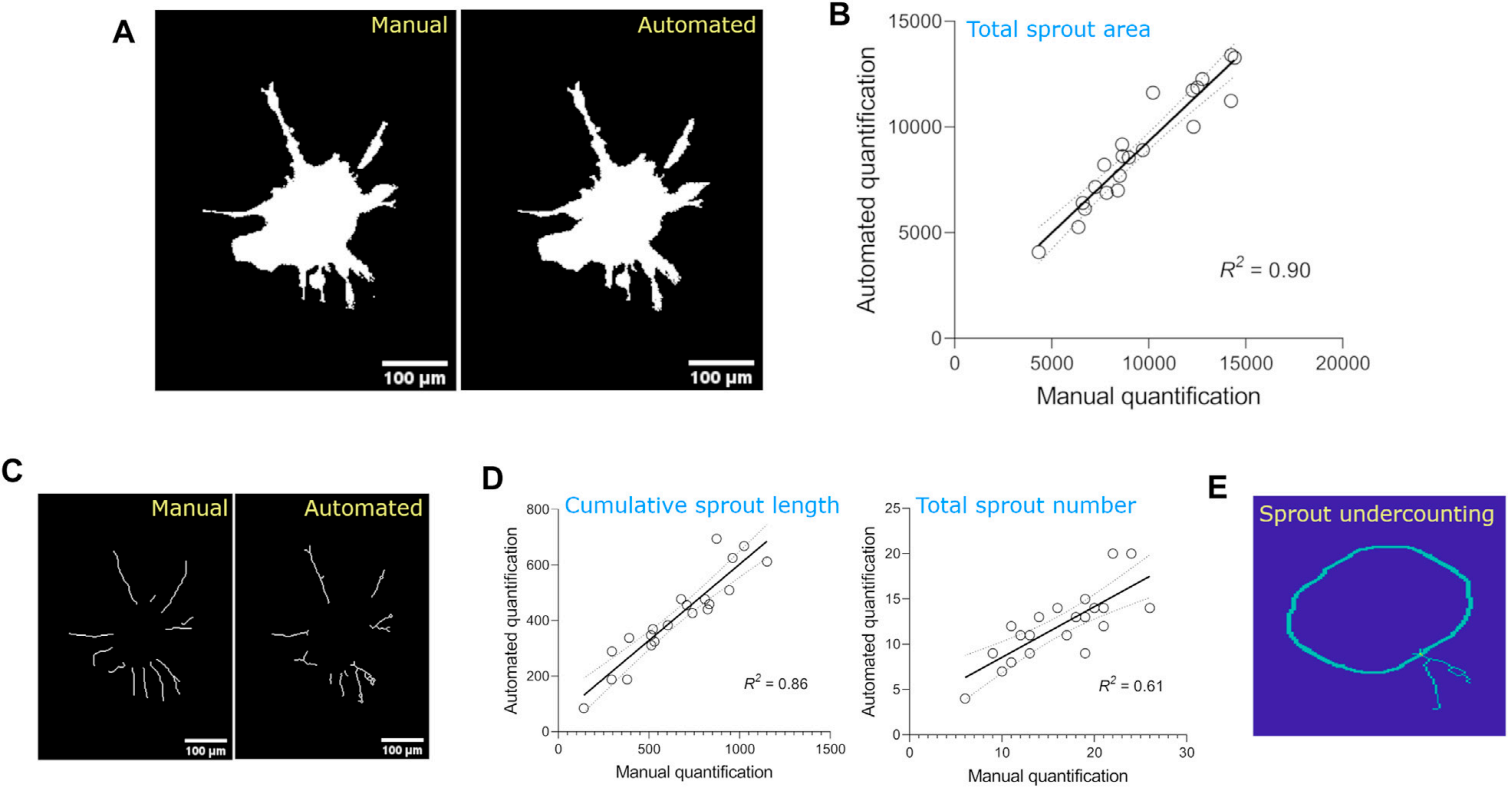
Comparison of sprouting parameters quantified from the optimization dataset using manual vs. automated methods. **(A)** Images of total spheroid masks detected using manual vs. automated methods. **(B)** Linear regression of total spheroid area quantified using manual vs. automated methods. For manual analysis, spheroid area was detected by thresholding images using the Triangle method on ImageJ and calculating the area of the obtained mask (*n* = 21 spheroids). Linear regressions are plotted with 95% confidence intervals. **(C)**Images of individual sprouts demarcated using manual vs. automated methods. **(D)** Linear regressions of cumulative sprout length and total number of sprouts quantified using manual vs. automated methods. For manual analysis, cumulative sprout length and number were measured using Simple Neurite Tracer plugin on ImageJ. Linear regressions are plotted with 95% confidence intervals. **(E)** Image demonstrating underestimation of sprout number detection by automated method.

Results from the automated quantification were then compared to manual quantification of the optimization dataset to determine the error/bias in the automated method; manual quantification was performed using ImageJ plugins. For simplicity, we compared three parameters: spheroid area, cumulative sprout length, and total number of sprouts. Manual and automated methods were well-correlated for total spheroid area (R^2^ = 90%, Figures 3A,B) and cumulative sprout length (R^2^ = 86%), but less correlated for total sprout number (R^2^ = 61%, Figures 3C,D), suggesting that the automated method underestimates the number of sprouts compared to manual quantification. Visual inspection of the output images from the automated method confirmed this idea, likely because of counting two sprouts as one (Figure 3E).

**FIGURE 4:**
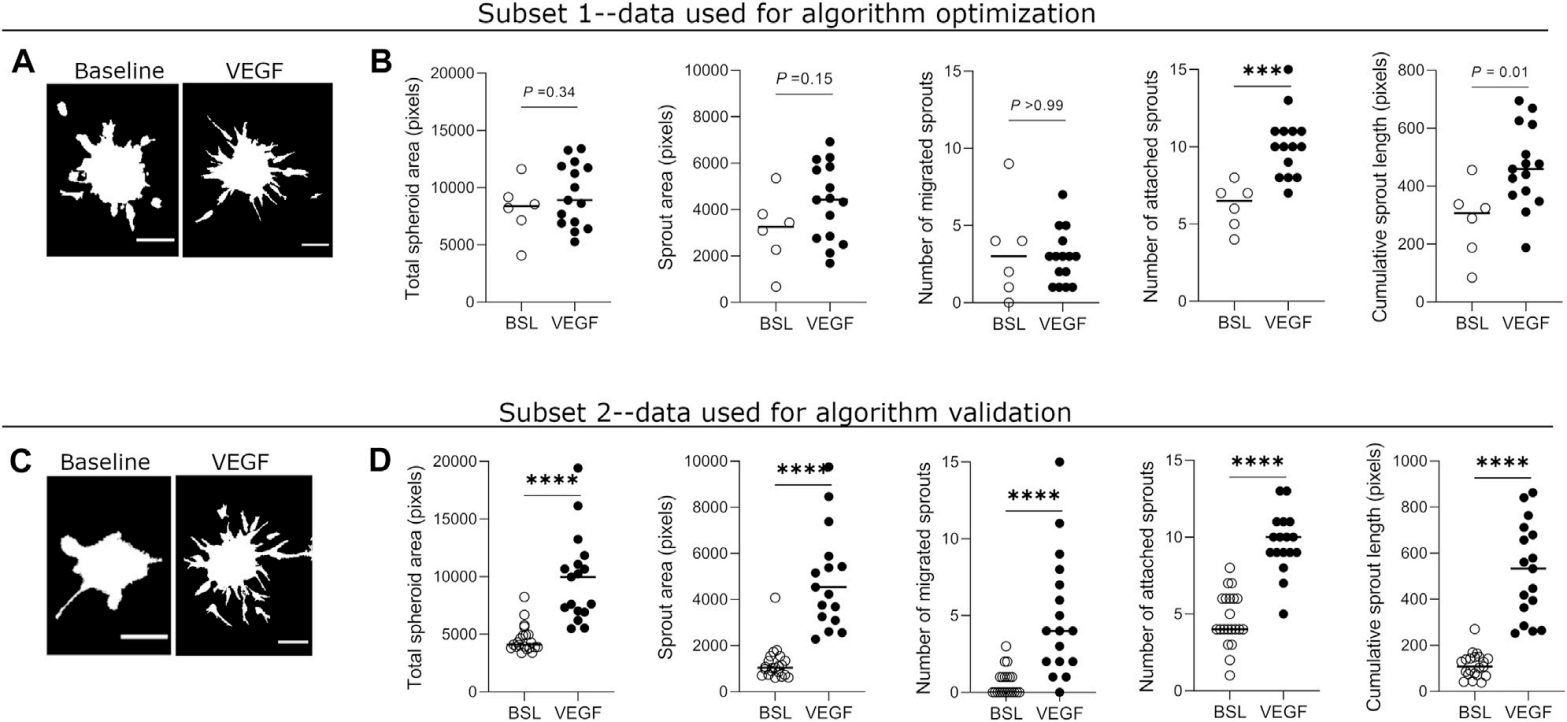
Quantification of sprouting parameters measured by automated method in optimization and validation datasets. **(A)** Representative binary masks of spheroid sprouting in baseline and VEGF-treated (25 ng/ml) conditions from the optimization dataset. VEGF = vascular endothelial growth factor. **(B)** Quantification of five sprouting parameters in untreated and VEGF-treated spheroids from the optimization dataset. **(C)** Binary mask images of spheroid sprouting in baseline and VEGF-treated (25 ng/ml) conditions from the validation dataset. **(D)** Quantification of sprouting parameters in untreated and VEGF-treated spheroids from validation dataset. Each data point represents value from one spheroid, and the horizontal line indicates the median value of the group. Data was collected from one biological experiment and divided into two subsets–one for optimization and one for validation of the automated method. Statistical significance for total spheroid area, sprout area, and cumulative sprout length was determined by Student’s t-test with Welch’s correction (unpaired, two-tailed, *α* = 0.05), while that for number of migrated sprouts and attached sprouts was determined by Mann-Whitney test (non-parametric, unpaired, two-tailed, *α* = 0.05). Adjusted *p*-values (corrected for multiple testing) are indicated. Scale bars = 100 μm.

To estimate the effect sizes and variance of sprouting parameter values, we quantified all parameters from a dataset in which HUVEC spheroids were treated with EGM-2 complete medium or the same medium supplemented with the angiogenesis inducer, vascular endothelial growth factor (VEGF, 25 ng/ml). In subset 1 of the data (Figures 4A,B), which was used to optimize the algorithm, we found significant differences between the two conditions in the number of attached sprouts and cumulative sprout length. The other three parameters did not result in significant differences, likely due to substantial variation in the parameter values and in the low sample size for baseline conditions (*n* = 6). Based on subset 1, we estimated that the effect sizes [Cohen’s d, (95% CI)] for the parameters were: small for total spheroid area [0.463, (−0.494 to 1.42)] medium for sprouting area [0.733, (−0.239 to 1.706)], small for migrated sprouts [−0.301, (−1.259 to 0.644)], and large for attached sprouts [1.543, (0.487 to 2.599)] and for cumulative sprout length [1.77, (0.683 to 2.858)] (Figures 4A,B).

We then re-ran the algorithm on subset 2 of the dataset containing a larger sample size (*n* = 21 for baseline; *n* = 17 for VEGF) to validate these results (Figures 4C,D). In subset 2 of the data, we found significant differences between the two conditions in all of the five measured parameters, despite substantial variation in nearly all parameter values. In this subset, estimated effect sizes [Cohen’s d, (95% CI)] for the parameters were large for all parameters: total spheroid area [1.942, (1.168 to 2.717)], sprouting area [2.134, (1.335 to 2.934)], migrated sprouts [1.745, (0.995 to 2.495)], attached sprouts [2.678, (1.8 to 3.557)] and cumulative sprout length [1.872, (1.107 to 2.638)] (Figures 4C,D). Together, these results suggest that attached sprouts and cumulative sprout length may be the most sensitive parameters when comparing differences in sprouting behavior between two conditions.

### 3.4 Automated Method Distinguishes Sprouting Behavior Among Pro- and Anti-Angiogenic Conditions

**FIGURE 5:**
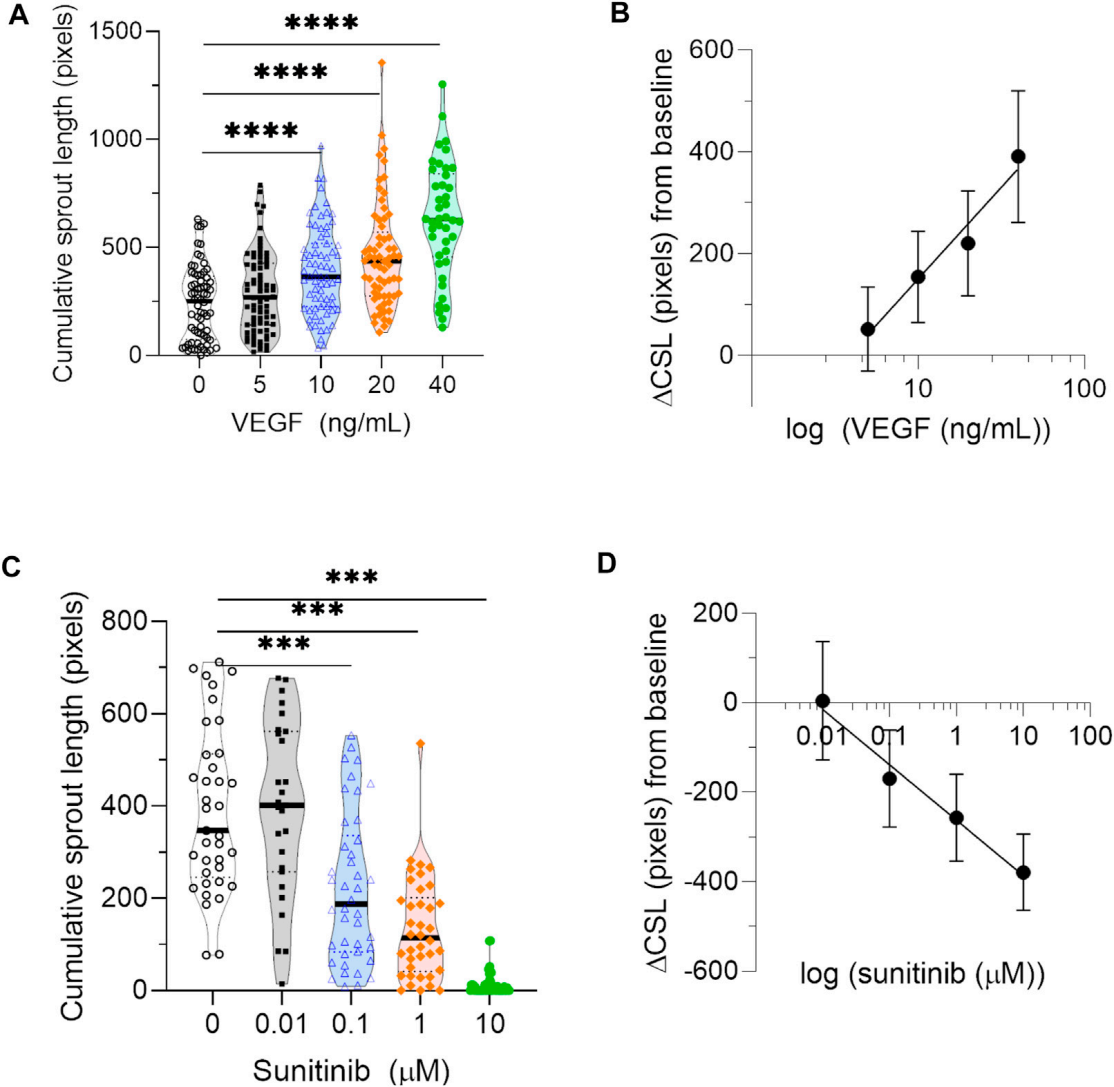
Sensitivity of automated quantification to pro- and anti-angiogenic compounds. **(A)** Quantification of cumulative sprout length measured in spheroids treated with a range of concentrations of the proangiogenic compound VEGF from three independent experiments. Each data point represents one spheroid. Statistical significance was determined by Browne-Forsythe one-way ANOVA, followed by multiple testing correction using Dunnett T3 (two-tailed, *α* = 0.05); asterisks designate adjusted *p*-values (corrected for multiple testing) as follows: ***p < 0.001. **(B)** The average difference in cumulative sprout length (ΔCSL) between VEGF-treated conditions and baseline, calculated for a range of VEGF concentrations. Data points represent average value in that group from all three biological repeats, and error bars represent the 95% confidence interval. **(C)** Quantification of cumulative sprout length measured in spheroids treated with a range of concentrations of the antiangiogenic inhibitor sunitinib from two independent experiments. Since sunitinib targets VEGF-mediated signaling by inhibiting VEGF receptor 2, sunitinib treatment was performed in the presence of 20 ng/ml VEGF for all conditions. Each data point represents one spheroid. Statistical significance was determined by Browne-Forsythe one-way ANOVA, followed by multiple testing correction using Dunnett T3 (two-tailed, *α* = 0.05); asterisks designate adjusted *p*-values (corrected for multiple testing) as follows: ****p < 0.0001. **(D)** The average difference in cumulative sprout length (ΔCSL) between sunitinib-treated conditions and baseline, calculated for a range of sunitnib concentrations. Data points represent average value in that group from both biological repeats, and error bars represent the 95% confidence interval.

Finally, to estimate the sensitivity of the automated algorithm and biological variation in the experimental setup, we measured changes in cumulative sprout length across a range of concentrations of pro- and anti-angiogenic compounds from independent experiments. Cumulative sprout length was significantly increased in HUVEC spheroids treated with increasing concentrations of angiogenic VEGF (≥10 ng/ml) in all three biological repeats (Figure 5A). Although absolute values of cumulative sprout length varied among experiments, differences in cumulative sprout length from baseline conditions were reproducible. Cumulative sprout length increased linearly from baseline conditions, when VEGF concentration was plotted on a log scale (Figure 5B). In contrast, cumulative sprout length significantly decreased in HUVEC spheroids treated with increasing concentrations of antiangiogenic sunitinib (≥100 nM) in both biological repeats (Figure 5C). Cumulative sprout length decreased linearly from baseline conditions, when sunitinib concentration was plotted on a log scale (Figure 5D).

## 4 Discussion

### 4.1 Automated Quantification of Angiogenic Sprouts From Spheroid-Based Assay

We developed and validated an automated algorithm for the quantification of fluorescent angiogenic sprouts from the HUVEC spheroid-based assay (AQuTAS) adapted to a 96-well plate format. The method relies on fluorescent labeling of HUVECs to enhance sprout detection within a 3-dimensional, semi-transparent fibrin gel. Once the method segments the total spheroid area, it generates a sprout skeleton used to calculate morphological parameters such as total number of sprouts, number of migrated and attached sprouts, as well as the cumulative sprout length. The algorithm detected known differences in sprouting parameters for HUVEC spheroids treated with proangiogenic treatment and was also sensitive to changes in sprouting parameters for HUVEC spheroids treated with varying concentrations of pro- and antiangiogenic treatments across different datasets. Our automated method therefore allows quantification of sprouting parameters from the HUVEC spheroid-based assay in a fast and reproducible way.

Among the sprouting parameters, cumulative sprout length is likely the most discriminative parameter for measuring differences in HUVEC sprouting behavior. In HUVEC spheroids treated with 25 ng/ml VEGF, we found significant increases in the cumulative sprout length compared to baseline. More importantly, the effect size of this parameter was large (>1.5) in response to one of the most potent angiogenic inducers (Ferrara and Henzel, 1989; Heiss et al., 2015; Eglinger et al., 2017). Although increases in other parameters such as total spheroid area and sprouting area were statistically significant when the sample size was increased (>15 spheroids/group), the effect sizes of these changes were relatively small (<1.0). Although previous studies have used a variety of metrics including cumulative sprout length (Heiss et al., 2015; Eglinger et al., 2017; Carpentier et al., 2020; Katsila et al., 2021), number of sprouts (Nakatsu et al., 2003; Newman et al., 2011; Eglinger et al., 2017; Carpentier et al., 2020), and sprouting area (Boettcher et al., 2010; Winters et al., 2016), our results indicate that the effect size of the sprouting parameter being measured is an important consideration when performing spheroid-based sprouting assay.

Given the biological variation of the sprouting assay, the range of sensitivity for algorithm detection is also an important factor to consider in the sprouting angiogenesis assay. Here, we measured the sensitivity of our algorithm by measuring changes in cumulative sprout length over a range of concentrations of pro- and antiangiogenic compounds. Our results confirm that the algorithm is sensitive to biological changes in cumulative sprout length. Similar to previous results (Heiss et al., 2015; Carpentier et al., 2020; Katsila et al., 2021), we found that VEGF concentrations ≥10 ng/ml increased cumulative sprout length by ≥ 1.6-fold, while sunitinib concentrations ≥100 nM decreased cumulative sprout length by ≥ 1.7-fold. Together, these findings demonstrate that the algorithm can detect biological changes that are at least 40% higher or lower than baseline. Based on the concentrations of drugs used in this study, we are unable to determine if the algorithm might be sensitive to changes less than 40%.

Although our method allows reproducible and sensitive quantification of cumulative sprout length over a large range of biological responses, it has a few limitations. First, it underestimates the number of sprouts by approximately half, compared to manual quantification. Nevertheless, although the absolute values of this parameter are underestimated, such an error is unlikely to be problematic for quantifying relative differences between groups. Second, since the method uses contrast to perform segmentation, it requires cells to be labeled fluorescently for enhanced contrast. Although fluorescence intensity can decrease with time and with cell proliferation, we do not expect that such decreases would affect the segmentation because it detects structures based on contrast rather than absolute intensity values. However, the method has not been validated on data from time-lapse experiments. We are working to create an update version of the method that can quantify sprouting structures from phase-contrast images, as it would eliminate the need for fluorescent labeling. Third, the method only quantifies the initial phase of sprouting angiogenesis. We were not able to measure the formation of stable lumens over time because of a shortage in fibroblasts, which have been shown to stabilize lumen formation in the fibrin bead assay (Newman et al., 2011). Thus, while our current setup and method are limited in the analysis of all steps of angiogenesis, they could instead be used to measure angiogenic sprouting induced by various tumor cells and other non-fibroblast cells in the future (Tatla et al., 2021).

In summary, we developed an automated algorithm to quantify sprouting parameters from the HUVEC spheroid-based assay. The algorithm, called AQuTAS, detects key biological differences in spheroids treated with pro- and antiangiogenic compounds. The optimized spheroid-sprouting assay and the algorithm provides researchers an alternative way to assess angiogenic sprouting, and these may be especially useful to measure angiogenic effects of non-fibroblast cells.

## Data Availability

The datasets and software generated for this study can be found in Zenodo (DOI: 10.5281/zenodo.6444392). Source code can be found in Github (https://github.com/p-kannan/aqutas). Additional inquiries can be directed to the corresponding author.

## References

[B1] Bertrand G. Malandain G. (1995). A Note on "Building Skeleton Models via 3-D Medial Surface/Axis Thinning Algorithms". Graphical Models Image Process. 57, 537–538. 10.1006/gmip.1995.1045

[B2] Boettcher M. Gloe T. de Wit C. (2010). Semiautomatic Quantification of Angiogenesis. J. Surg. Res. 162, 132–139. 10.1016/j.jss.2008.12.009 19345375

[B3] Carpentier G. Berndt S. Ferratge S. Rasband W. Cuendet M. Uzan G. (2020). Angiogenesis Analyzer for ImageJ - A Comparative Morphometric Analysis of "Endothelial Tube Formation Assay" and "Fibrin Bead Assay". Sci. Rep. 10, 1–13. 10.1038/s41598-020-67289-8 32665552 PMC7360583

[B4] Dos Santos S. N. Sheldon H. Pereira J. X. Paluch C. Bridges E. M. El-Cheikh M. C. (2017). Galectin-3 Acts as an Angiogenic Switch to Induce Tumor Angiogenesis via Jagged-1/Notch Activation. Oncotarget 8, 49484–49501. 10.18632/oncotarget.17718 28533486 PMC5564783

[B5] Eglinger J. Karsjens H. Lammert E. (2017). Quantitative Assessment of Angiogenesis and Pericyte Coverage in Human Cell-Derived Vascular Sprouts. Inflamm. Regen. 37, 2. 10.1186/s41232-016-0033-2 29259701 PMC5725907

[B6] Favara D. M. Liebscher I. Jazayeri A. Nambiar M. Sheldon H. Banham A. H. (2021). Elevated Expression of the Adhesion GPCR ADGRL4/ELTD1 Promotes Endothelial Sprouting Angiogenesis without Activating Canonical GPCR Signalling. Sci. Rep. 11, 8870–8913. 10.1038/s41598-021-85408-x 33893326 PMC8065136

[B7] Feng X. Tonnesen M. G. Mousa S. A. Clark R. A. (2013). Fibrin and Collagen Differentially but Synergistically Regulate Sprout Angiogenesis of Human Dermal Microvascular Endothelial Cells in 3-dimensional Matrix. Int. J. Cel Biol 2013, 231279. 10.1155/2013/231279 PMC3657431 23737792

[B8] Ferrara N. Henzel W. J. (1989). Pituitary Follicular Cells Secrete a Novel Heparin-Binding Growth Factor Specific for Vascular Endothelial Cells. 1989. Biochem. Biophys. Res. Commun. 425, 540–547. 10.1016/j.bbrc.2012.08.021 22925671

[B9] Heiss M. Hellström M. Kalén M. May T. Weber H. Hecker M. (2015). Endothelial Cell Spheroids as a Versatile Tool to Study Angiogenesis *In Vitro* . FASEB J. 29, 3076–3084. 10.1096/fj.14-267633 25857554

[B10] Kannan P. Schain M. Kretzschmar W. W. Weidner L. Mitsios N. Gulyás B. (2016). An Automated Method Measures Variability in P-Glycoprotein and ABCG2 Densities across Brain Regions and Brain Matter. J. Cereb. Blood Flow Metab. 37, 2062–2075. 10.1177/0271678X16660984 27488911 PMC5464701

[B11] Katsila T. Chasapi S. A. Gomez Tamayo J. C. Chalikiopoulou C. Siapi E. Moros G. (2021). Three-dimensional Cell Metabolomics Deciphers the Anti-angiogenic Properties of the Radioprotectant Amifostine. Cancers (Basel) 13. 10.3390/cancers13122877 PMC8230228 34207535

[B12] Korff T. Augustin H. G. (1998). Integration of Endothelial Cells in Multicellular Spheroids Prevents Apoptosis and Induces Differentiation. J. Cel Biol 143, 1341–1352. 10.1083/jcb.143.5.1341 PMC2133072 9832561

[B13] Longair M. H. Baker D. A. Armstrong J. D. (2011). Simple Neurite Tracer: Open Source Software for Reconstruction, Visualization and Analysis of Neuronal Processes. Bioinformatics 27, 2453–2454. 10.1093/bioinformatics/btr390 21727141

[B14] Nakatsu M. N. Davis J. Hughes C. C. (2007). Optimized Fibrin Gel Bead Assay for the Study of Angiogenesis. J. Vis. Exp. 186, 186–193. 10.3791/186 PMC2570172 18978935

[B15] Nakatsu M. N. Hughes C. C. (2008). An Optimized Three-Dimensional *In Vitro* Model for the Analysis of Angiogenesis. Methods Enzymol. 443, 65–82. 10.1016/S0076-6879(08)02004-1 18772011

[B16] Nakatsu M. N. Sainson R. C. Aoto J. N. Taylor K. L. Aitkenhead M. Pérez-del-Pulgar S. (2003). Angiogenic Sprouting and Capillary Lumen Formation Modeled by Human Umbilical Vein Endothelial Cells (HUVEC) in Fibrin Gels: The Role of Fibroblasts and Angiopoietin-1. Microvasc. Res. 66, 102–112. 10.1016/S0026-2862(03)00045-1 12935768

[B17] Nehls V. Herrmann R. (1996). The Configuration of Fibrin Clots Determines Capillary Morphogenesis and Endothelial Cell Migration. Microvasc. Res. 51, 347–364. 10.1006/mvre.1996.0032 8992233

[B18] Newman A. C. Nakatsu M. N. Chou W. Gershon P. D. Hughes C. C. (2011). The Requirement for Fibroblasts in Angiogenesis: Fibroblast-Derived Matrix Proteins Are Essential for Endothelial Cell Lumen Formation. Mol. Biol. Cel 22, 3791–3800. 10.1091/mbc.E11-05-0393 PMC3192859 21865599

[B19] Nowak-Sliwinska P. Alitalo K. Allen E. Anisimov A. Aplin A. C. Auerbach R. (2018). Consensus Guidelines for the Use and Interpretation of Angiogenesis Assays. Angiogenesis 21, 425–532. 10.1007/s10456-018-9613-x 29766399 PMC6237663

[B20] Potente M. Gerhardt H. Carmeliet P. (2011). Basic and Therapeutic Aspects of Angiogenesis. Cell 146, 873–887. 10.1016/j.cell.2011.08.039 21925313

[B21] Sun L. Liang C. Shirazian S. Zhou Y. Miller T. Cui J. (2003). Discovery of 5-[5-Fluoro-2-Oxo-1,2- Dihydroindol-(3z)-Ylidenemethyl]-2,4- Dimethyl-1h-Pyrrole-3-Carboxylic Acid (2-diethylaminoethyl)amide, a Novel Tyrosine Kinase Inhibitor Targeting Vascular Endothelial and Platelet-Derived Growth Factor Receptor Tyrosine Kinase. J. Med. Chem. 46, 1116–1119. 10.1021/jm0204183 12646019

[B22] Tatla A. S. Justin A. W. Watts C. Markaki A. E. (2021). A Vascularized Tumoroid Model for Human Glioblastoma Angiogenesis. Sci. Rep. 11, 19550–19559. 10.1038/s41598-021-98911-y 34599235 PMC8486855

[B23] Tetzlaff F. Fischer A. (2018). Human Endothelial Cell Spheroid-Based Sprouting Angiogenesis Assay in Collagen. Bio-Protocol 8, 1–7. 10.21769/bioprotoc.2995 PMC8328656 34395793

[B24] Winters L. Thambi N. Andreev J. Kuhnert F. (2016). Evaluation of Angiogenesis Inhibitors Using the HUVEC Fibrin Bead Sprouting Assay. Bio-Protocol 6, 1–9. 10.21769/bioprotoc.1947 27642615

