# Supplementary material for: An Automated Quantification Tool for Angiogenic Sprouting From Endothelial Spheroids
Source: Front Pharmacol. 2022 Apr 27;13:883083. doi: 10.3389/fphar.2022.883083 (PMC9093605; doi:10.3389/fphar.2022.883083)
Supplement: Supplementary file 1 [file DataSheet1.PDF]

Supplementary Material

1 SUPPLEMENTARY TABLES AND FIGURES

1.1 Figures

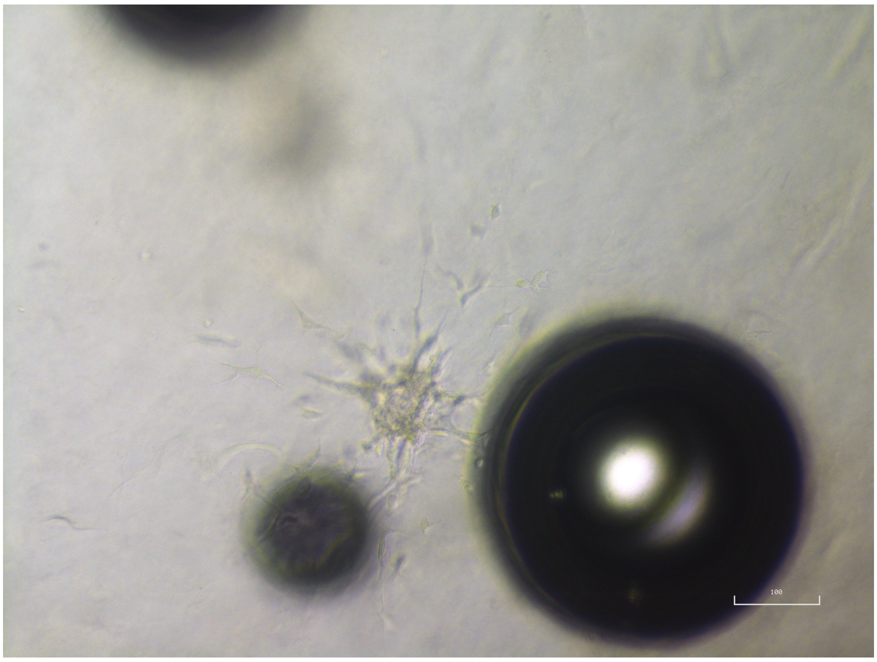

Figure S1. Phase contrast image of HUVEC spheroid sprouting in collagen gel taken 24 h after embedding. Note the numerous bubbles that result from pipetting a viscous solution. HUVEC = human umbilical vein endothelial cells

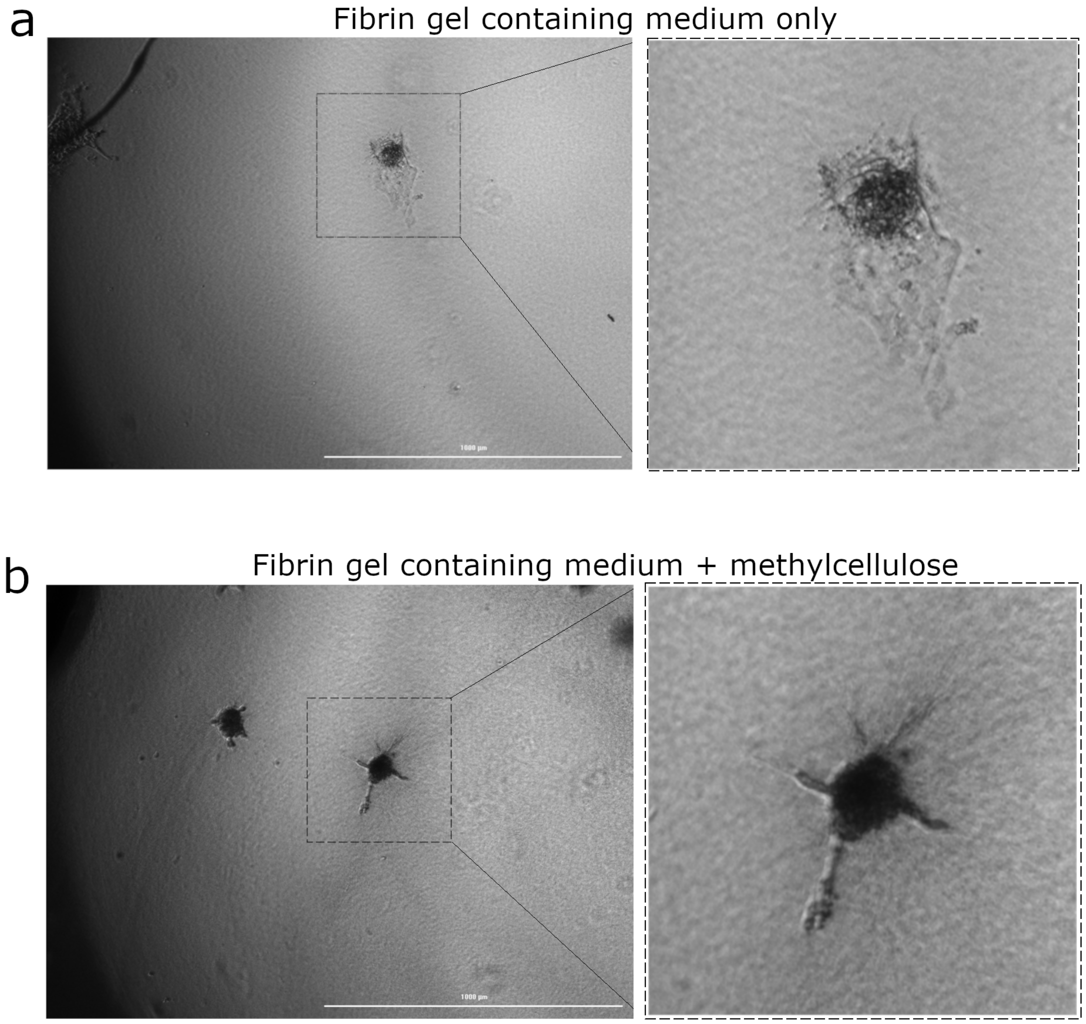

Figure S2. Addition of methylcellulose helps keep HUVEC spheroids suspended in fibrin gel. **(a)** Phase contrast image showing a HUVEC spheroid that has attached to the bottom of the well, 24 h after embedding in fibrin gel made in medium. **(b)** Phase contrast image showing a HUVEC spheroid that has remains suspended in gel, 24 h after embedding in fibrin gel made in medium containing 40% methylcellulose.

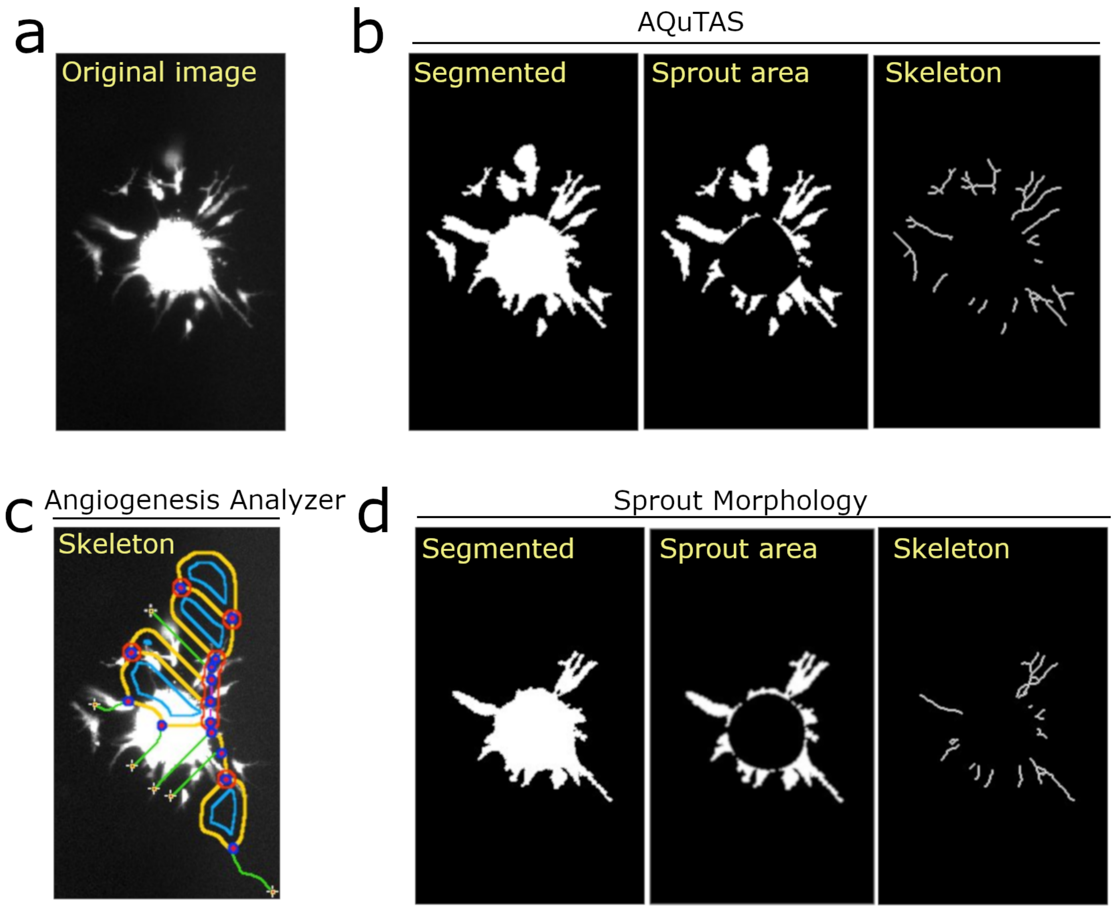

Figure S3. Visual comparison of spheroid sprouting, as quantified by three automated quantification tools for angiogenesis sprouting. **(a)** Fluorescence microscope image of spheroid sprout. **(b)** Segmentation and skeletonization output of the spheroid sprout in **(a)** using AQuTAS, developed for quantification of sprouts from HUVEC spheroids. **(c)** Skeletonization output of the spheroid sprout in **(a)** using Angiogenesis Analyzer, which was developed for quantification of sprouts from the fibrin bead assay. **(d)** Segmentation and skeletonization output of the spheroid sprout in **(a)** using Sprout Morphology, which was developed for quantification of sprouts from the fibrin bead assay.
